# Assessing the diagnostic accuracy of cardisiography in the diagnosis of coronary heart disease: CSG-DR study (A pilot study)

**DOI:** 10.3389/fcvm.2026.1845141

**Published:** 2026-07-21

**Authors:** Rafael A. Guillén-Marmolejos, Rodolfo A. Núñez-Musa, Alberto J. Núñez-Selles, Ana J. Santos-Rondón, Miguel Arias-Ceballos, the CSG Group

**Affiliations:** 1 Centro de Diagnóstico Especializado (CEDISA), Santo Domingo, República Dominicana.; 2 Contract Research Organization DR (CREODR), Santo Domingo, República Dominicana; 3 Research Division, Universidad Nacional “Pedro Henríquez Ureña” (UNPHU), Santo Domingo, Dominican Republic

**Keywords:** cardisiography, coronary artery disease, coronary computerized tomography angiography, diagnostic accuracy, electrocardiography

## Abstract

**Background:**

Early and accurate detection of coronary artery disease (CAD) remains a challenge in primary care, particularly in low- and middle-income countries where access to advanced diagnostic imaging is limited. A resting electrocardiogram (ECG) is widely available but has low sensitivity for detecting ischemia. Cardisiography (CSG), an artificial intelligence–enhanced vectorcardiography technique, offers a promising non-invasive alternative.

**Methods:**

This single-center, prospective, double-blinded pilot study enrolled 104 patients aged 40 and above with suspected CAD referred for coronary computerized tomography angiography (CCTA). All participants underwent ECG, CSG, and CCTA as the reference standard. Diagnostic accuracy was assessed using the CAD-RADS classification.

**Results:**

CCTA identified coronary lesions classified as CAD-RADS 1–3 in 29 patients (12, 11, and 6, respectively). CSG achieved an overall sensitivity of 96.5% (28/29), compared with 6.9% (2/29) for ECG. Sensitivity was 100% for CAD-RADS 1 and 3, and 91% for CAD-RADS 2. Diagnostic accuracy metrics are reported with 95% confidence intervals.

**Conclusion:**

In this pilot referral cohort, CSG demonstrated higher sensitivity than resting ECG for detecting coronary plaque. These preliminary findings suggest CSG may serve as a triage tool before CCTA or cardiology referral. Larger, multicenter studies are needed to validate its role and determine clinical utility.

**Clinical Trial Registration:**

https://conabios.gob.do/reglamento, identifier 034-2023.

## 1 Introduction

Cardiovascular disease (CVD) remains the leading cause of death worldwide, accounting for an estimated 18.5 million fatalities each year (1). Despite a historical decline in age-standardized CVD mortality rates over recent decades, a paradox has emerged since 2010: mortality rates have either increased or stagnated in many regions, while progress has stalled in others where the rates have leveled off (1). A comprehensive analysis of CVD mortality reduction in a North American study between 1980 and 2000 identified two primary contributing factors: the implementation of highly specific, resource-intensive interventions, and the widespread adoption of more universal prevention strategies (2). The effectiveness of the first group of interventions is often restricted due to their high cost and technological complexity, which limits their accessibility in many low- and middle-income countries (LMICs). Furthermore, the rising prevalence of risk factors such as obesity and type 2 diabetes mellitus counteract the downward trends in CVD mortality. As a result, the risk of severe coronary vascular damage remains a significant concern (2, 3).

Coronary artery disease (CAD), also known as ischemic heart disease, is the most common form of CVD and the leading cause of death worldwide, surpassing all other conditions, including cancer (4). The diagnostic process for CAD in primary care usually includes a clinical history, a physical examination, and a resting 12-lead electrocardiogram (ECG). While the resting ECG is widely accessible, it has limited sensitivity and specificity for detecting ischemia, especially in its early or atypical forms (5–7). As a result, patients are referred for more advanced tests such as stress testing and coronary computerized tomography angiography (CCTA). These procedures are resource-intensive and often inaccessible in low-resource settings, where approximately 75% of global CVD deaths occur (8). This creates a diagnostic gap that contributes to delayed treatment and poorer outcomes.

The limitations of traditional methods for diagnosing CVD are well-documented (9). Independent ECG parameters, such as ST-T changes or Q waves, are often poor predictors of CVD. Even when these parameters are combined, they yield a positive diagnosis rate of only 71% (10). The maximum reported sensitivity for standard and exercise ECGs is 68%, while the specificity for exercise ECG is 77% (11). Cardisiography (CSG) is an artificial intelligence–enhanced vectorcardiography technique that analyzes subtle electrical changes associated with myocardial hypoperfusion and structural alterations. By capturing three-dimensional cardiac electrical activity and applying machine-learning algorithms, CSG may detect abnormalities not visible on standard ECG. Early studies have suggested promising diagnostic accuracy, but validation in diverse populations remains necessary (11). The CSG-DR Study was designed as a pilot investigation to evaluate the diagnostic accuracy of CSG compared with resting ECG, using CCTA as the reference standard, in patients with suspected CVD in the Dominican Republic. Our objective is to provide preliminary evidence on the sensitivity and specificity of CSG in this referral population, while acknowledging the limitations of sample size, single-center design, and the predominance of non-obstructive lesions.

## 2 Materials and methods

### 2.1 Study protocol

This study was designed as a prospective, double-blinded, pilot investigation conducted at a single center in Santo Domingo, Dominican Republic (12). Ethical approval was obtained from the national regulatory authority (Registry No. 034-2023 CONABIOS).

### 2.2 Population

We enrolled 104 patients referred for coronary computerized tomography angiography (CCTA) due to suspected CVD. Inclusion criteria were age ≥40 years, clinical suspicion of CAD, and indication for CCTA. Exclusion criteria included age <40, pregnancy or puerperium, withdrawal of consent, or refusal to undergo CCTA. Three participants aged 20–40 represented protocol deviations and are documented explicitly in Table 1.

**Table 1:** Baseline demographic and clinical characteristics of the study cohort (*n* = 100).

Characteristic	Group	*n*	Group	*n*	Group	*n*	Group	*n*	Group	*n*
Age	20–40 y	3	42–50 y	23	51–60 y	25	61–70 y	28	71–80 y	21
Skin	White	44	Mixed	42	Asian	10				
Gender	Male	47	Female	53						
Weight	Mean (kg)	79.3	S.D.	15.9						
Height	Mean (cm)	157.8	S.D.	13.1						
BMI	Mean (kg/m^2^)	31.8	S.D.	2.7						

Comorbidities	*n*	Risk factors	*n*
Diabetes mellitus type 2	12	Arrhythmia	17
Arterial Hypertension	65	Angina	13
CVD backgrounds	24	CVD	70
Smoking	14	ASCVD ≤10%	92
Exercise >2.5 h/week	11	ASCVD 11%–20%	8

Three participants aged 20–40 years were retained as protocol deviations because they met all other inclusion criteria and completed CCTA, allowing their data to contribute to diagnostic accuracy estimates.

S.D., standard deviation; BMI, body mass index; CVD, cardiovascular disease; ASCVD, atherosclerotic risk calculator.

### 2.3 Blinding

CCTA readers were blinded to CSG and ECG results. ECG interpretation was performed independently, blinded to CSG and CCTA. CSG outputs were generated and locked before comparison with CCTA.

### 2.4 Electrocardiography (ECG)

Resting 12-lead ECGs were performed using standardized equipment and reference ranges. An ECG was considered positive for CAD if ischemic changes were present (ST-segment deviation or pathological T-wave inversion). Conduction abnormalities, rhythm disturbances, or axis deviations not related to ischemia were not classified as CAD-positive.

### 2.5 Cardisiography (CSG)

**Figure 1:**
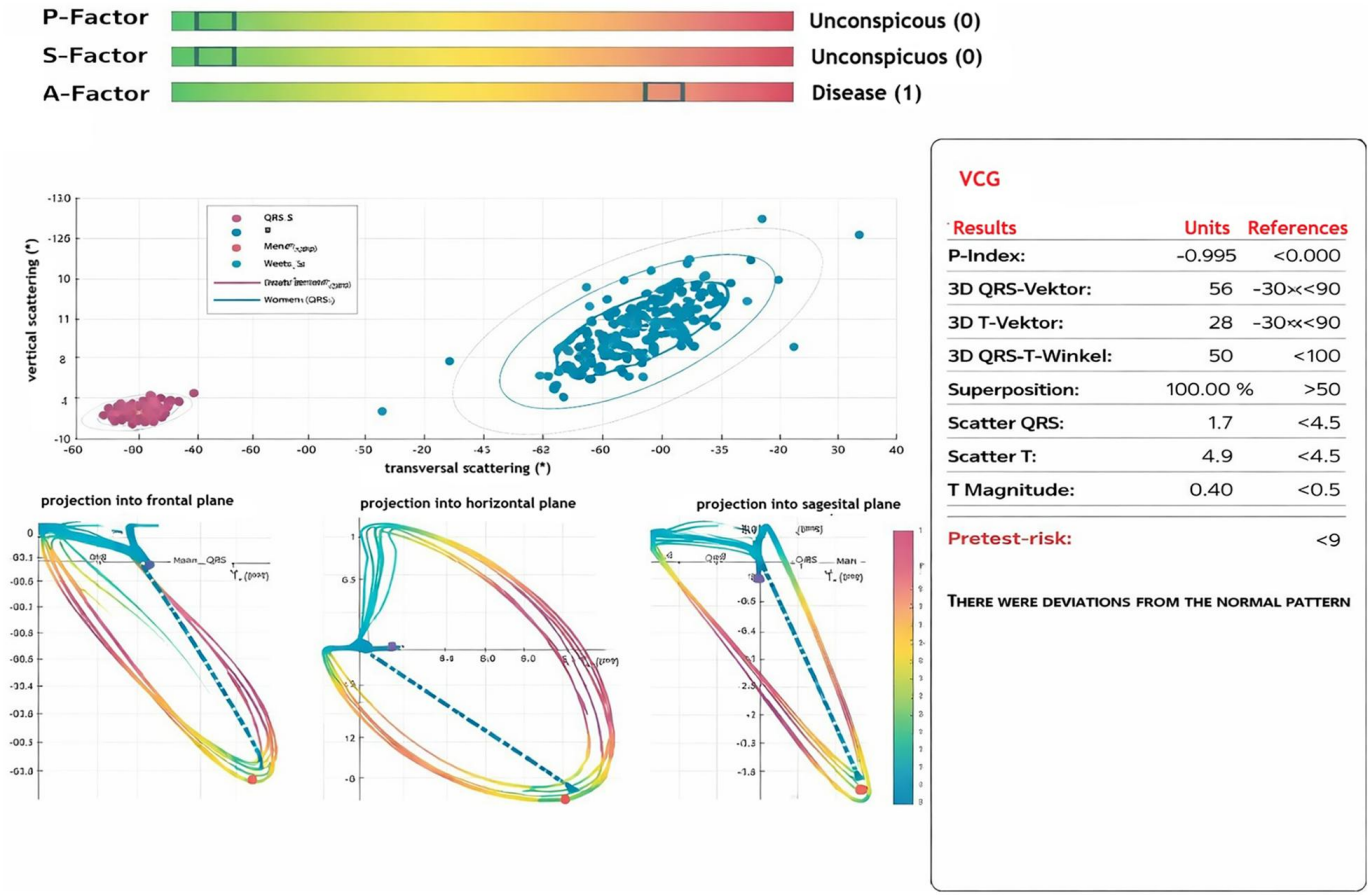
Typical CSG report. The upper part shows the graphical results for factors P (perfusion), S (structure), and A (arrhythmia), numerical results of these factors' calculations are shown in the right box, and vectorcardiographic images of each factor are shown in the left box.

CSG was performed at rest using the Cardisio system (Cardisio GmbH, Frankfurt, Germany). The method acquires three-dimensional vectorcardiographic data and applies a neural-network (AI) algorithm to generate automated scores for perfusion, structural, and arrhythmic risk. The algorithm generated traffic-light outputs (green, yellow, orange, red) for perfusion (P), structure (S), and arrhythmia (A) factors. A CSG test was considered positive if any factor reached the “red” category. This rule was pre-specified before comparison with CCTA. As seen in Figure 1, the reading is a quick visual impression.

### 2.6 Computerized catheterized coronary tomography angiography (CCTA)

CCTA studies were performed on a 64-detector Philips Ingenuity scanner (Philips Healthcare) in accordance with standard protocols. Coronary lesions were classified according to CAD-RADS. For this study, “disease-positive” was defined as CAD-RADS 1–3. Results are reported separately for non-obstructive (RADS 1–2) and obstructive (RADS 3).

### 2.7 Statistical analysis

Diagnostic accuracy metrics (sensitivity, specificity, positive predictive value, negative predictive value, and accuracy) were calculated with 95% confidence intervals. Likelihood ratios and Cohen's kappa were included to assess agreement. McNemar's test was applied to discordant pairs in the full cohort. BMI was calculated for each participant and reported as the mean ± SD.

## 3 Results

### 3.1 Population

**Figure 2:**
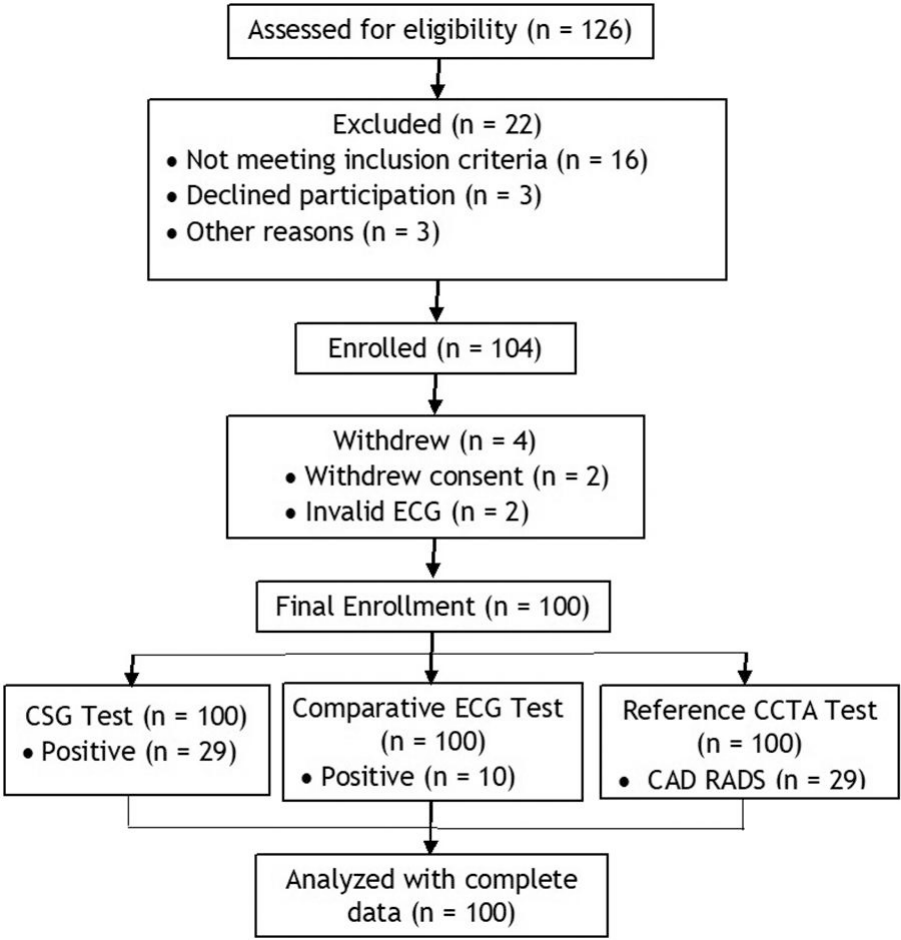
STARD flow diagram summarizing participant screening, enrollment, diagnostic testing, and analysis in the CSG-DR pilot study. The diagram details the number of individuals assessed for eligibility, excluded for reasons, enrolled, tested by each modality (CSG, Cardisiography; ECG, electrocardiography; and CCTA, coronary computed tomography angiography), and included in the final analysis.

Of 104 enrolled patients, 4 were excluded (withdrew consent or had invalid scans), leaving 100 analyzed (see Figure 2). Baseline characteristics are presented in Table 1, including age, sex, cardiovascular risk factors, prior CVD, and indication for CCTA. BMI was calculated for each participant and reported as the mean ± SD. The cohort included slightly more females (52.9%) than males (47.1%). The mean age was 61 years, with 65.5% having hypertension, 12.5% diabetes, 14.4% smoking history, and 24% prior CVD.

### 3.2 ECG findings

The resting ECG showed mostly normal values. Minor abnormalities included axis deviation (2 cases), arrhythmias (3), nonspecific T-wave changes (3), and ST-segment deviation (1). Only ischemic changes (ST deviation, pathological T-wave inversion) were classified as CAD-positive. Conduction or rhythm abnormalities were not considered CAD-positive. Table 2 summarizes the electrocardiographic findings in the study population.

**Table 2:** Electrocardiographic findings of the cohort (*n* = 100) for the CSG-DR study.

Parameter	Observed		Notes
	Normal	Deviated	
Axis	98	2	–
Rhytm	97	3	–
Heart rate	96	4	Tachycardia or bradycardia (Mild)
*P* wave	99	1	Possible atrial enlargement
QRS	98	2	Compatible with mild block (Widened)
ST	99	1	Possible mild ischemia
T wave	97	3	Inverted. Non-specific alterations
Total			90 patients showed no abnormality

### 3.3 Cardisiography (CSG)

CSG traffic-light outputs are summarized in Table 3. Most patients were “green” for perfusion, structure, and arrhythmia. A CSG test was considered positive if any factor reached “red.” This yielded 29 positives, consistent with CCTA results.

**Table 3:** Results of the CSG evaluation of the cohort (*n* = 100) for the CSG-DR study.

Factor	No. patients				
	Green	Yellow	Orange	Red	Total
P	60	12	11	17	100
S	74	11	8	7	100
A	90	2	3	5	100

### 3.4 Computerized catheterized coronary tomography angiography (CCTA)

CCTA identified 29 patients with CAD-RADS 1–3 lesions (12, 11, and 6, respectively). Table 4 presents the distribution of CAD-RADS classes.

**Table 4:** CCTA findings in the cohort (*n* = 100) for the CSG-DR study.

Class	No. Patients	Sensitivity (%)
CAD-RADS 1	12	100
CAD-RADS 2	11	91
CAD-RADS 3	6	100
TOTAL	29	96.5

### 3.5 Diagnostic accuracy

CSG achieved sensitivity of 96.5% (95% CI: 28–29), specificity of 93.1% (95% CI: 27–29), PPV 87.5% (95% CI: 71–96), NPV 98.5% (95% CI: 91–100), and overall accuracy 94% (95% CI: 87–98). ECG achieved sensitivity of 6.9% (95% CI: 2–22), specificity of 97.2% (95% CI: 90–99), PPV 50% (95% CI: 15–85), NPV 72.6% (95% CI: 62–81), and accuracy 71% (95% CI: 61–79). Results are reported separately for CAD-RADS 1–2 (non-obstructive) and CAD-RADS 3 (obstructive). 8/10 ECG abnormal results did not match with CAD-RAS lesions from CCTA, whereas CSG showed 1 false positive. Likelihood ratios and Cohen's kappa were included. Diagnostic performance is summarized in Table 5.

**Table 5:** Diagnostic accuracy of CSF and ECG compared to CCTA (reference).

Test vs. CCTA	Sensitivity % (95% CI)	Specificity % (95% CI)	PPV % (95% CI)	NPV % (95% CI)	Accuracy % (95% CI)	LR+	LR–	Κ
CSG	96.5 (82–100)	98.6 (86–100)	96.6 (74–98)	98.6 (91–100)	98.0 (89–99)	13.6	0.04	0.88
ECG	6.9 (2–22)	97.2 (90–99)	50 (15–85)	72.6 (62–81)	71 (61–79)	2.5	0.95	0.12

CCTA, computerized coronary tomography angiography; CSG, cardisiography; ECG, electrocardiogram; CI, confidence interval; PPV, positive predictive value; NPV, negative predictive value; LR, likelihood ratio; Κ, Cohen's kappa.

## 4 Discussion

This pilot study evaluated the diagnostic accuracy of Cardisiography (CSG) compared with resting ECG, using CCTA as the reference standard, in patients referred for suspected cardiovascular disease. Our main finding was that CSG achieved markedly higher sensitivity than ECG for detecting coronary plaque (Table 5). Specifically, CSG sensitivity was 96.5% (95% CI: 82–100), while ECG sensitivity was only 6.9% (95% CI: 2–22). Specificity was high for both tests, but predictive values differed substantially. Results differed by CAD-RADS category. As shown in Table 6, most positives were non-obstructive (CAD-RADS 1–2), with only six patients classified as obstructive (CAD-RADS 3). Thus, the high sensitivity of CSG reflects the detection of any plaque, not necessarily clinically significant obstructive CAD.

**Table 6:** Stratified CSG performance by CAD-RADS score (CCTA + subgroup, *n* = 29).

CAD-RADS category	CCTA positive (*n*)	CSG true positives (TP)	Sensitivity (%)
1	12	12	100.0
2	11	10	90.9
3	6	6	100.0
Total (1–3)	29	28	96.5

CAD, cardiovascular disease; CCTA, computerized coronary tomography angiography; CSG: cardisiography.

This distinction is critical: resting ECG is designed to detect ischemia or infarction, not non-obstructive plaque. Therefore, the contrast between CSG and ECG must be interpreted cautiously. ECG remains indispensable for the evaluation of arrhythmias, conduction disorders, and cardiomyopathies, while CSG may serve as a complementary triage tool before CCTA or cardiology referral.

International studies reported generally high sensitivity for CSG in detecting CAD. For example, Braun et al*. (*11) (multicenter, *n* = 595) found that CSG identified CAD with a sensitivity of 90%–97% (97% in men, 90% in women) and a specificity of 74%–76% (11). In a validation study at the Sana Heart Center (*n* = 106). In contrast, one nuclear-medicine study (*n* = 88) showed CSG had 100% sensitivity and 98% negative predictive value (NPV) for myocardial ischemia (everyone with disease was detected), albeit lower specificity (57%) (13). Together, these findings indicate CSG strongly favors detection over false negatives. Its NPV is exceptionally high (90%–98% in these studies as in ours), meaning a normal CSG test reliably excludes significant CAD. Specificity is more variable (60%–90%), so some false positives may occur.

Additionally, Aydar et al. (14) (*n* = 241) and Rudland et al. (15) (*n* = 628, asymptomatic primary-care cohort) consistently showed CSG excels at identifying individuals without significant CAD (high NPV) and has very high sensitivity in most screened populations. Its moderate specificity means that some healthy individuals may screen positive and require confirmatory imaging. Nonetheless, overall diagnostic accuracy (area under ROC curves) tends to exceed conventional ECG screening.

This study has several limitations. The sample size was small, single-center, and non-consecutive, limiting generalizability. Confidence intervals around subgroup estimates are wide, and no CAD-RADS 4–5 cases were included. Blinding procedures and positivity thresholds have been clarified, but external validation of the proprietary CSG algorithm remains necessary.

In conclusion, CSG demonstrated promising diagnostic accuracy for detecting coronary plaque in this referral cohort. Rather than suggesting that CSG could replace ECG or revolutionize screening, we position it as a potential triage tool that warrants validation in larger, multicenter studies.

## 5 Conclusion

In this pilot referral cohort, Cardisiography (CSG) demonstrated higher sensitivity than resting ECG for detecting coronary plaque identified by CCTA. These findings suggest that CSG may serve as a complementary triage tool before CCTA or cardiology referral. However, given the small sample size, single-center design, and predominance of non-obstructive lesions, the results should be interpreted cautiously. Larger, multicenter studies are required to validate these preliminary observations and to determine the clinical utility of CSG in diverse populations.

## Data Availability

The original contributions presented in the study are included in the article/Supplementary Material, further inquiries can be directed to the corresponding author.

## CSG-DR Group

Tulio A. Ramírez-Paniagua, Carmen L. Rodríguez-Díaz, Máxima Méndez-Castillo, Rosario A. Rodríguez-Feliz, Mildred I. Ureña-Rivera, Nilvia Castillo-Presbot, and Gerald Sarante-Brand.

## References

[1] Roth GA Mensah GA Fuster V. The global burden of cardiovascular diseases and risks. J Am Coll Cardiol. (2020) 76(25):2980–1. 10.1016/j.jacc.2020.11.021 33309174

[2] Ford ES Ajani UA Croft JB Critchley JA Labarthe DR Kottke TE. Explaining the decrease in U.S. Deaths from coronary disease, 1980–2000. Obstet Gynecol Surv. (2007) 62(10):664–5. 10.1097/01.ogx.0000282009.19829.c3 17554120

[3] Fox CS Coady S Sorlie PD D’Agostino RB Pencina MJ Vasan RS. Increasing cardiovascular disease burden due to diabetes mellitus. Circulation. (2007) 115(12):1544–50. 10.1161/CIRCULATIONAHA.106.658948 17353438

[4] Mozaffarian D Benjamin EJ Go AS Arnett DK Blaha MJ Cushman M. Heart disease and stroke statistics−2015 update: a report from the American Heart Association. Circulation. (2015) 131(4):e29–322. 10.1161/CIR.0000000000000152 25520374

[5] Lu DY Yang AC Cheng HM. Heart rate variability is associated with exercise capacity in patients with cardiac syndrome X. PLoS One. (2016) 11(1):e0144936. 10.1371/journal.pone.0144935 PMC4727925 26812652

[6] Aronow WS. Silent MI. Prevalence and prognosis in older patients diagnosed by routine electrocardiograms. Geriatrics. (2003) 58(1):24–40. Available online at: https://europepmc.org/article/med/12545670 12545670

[7] Mahmoodzadeh S Moazenzadeh M Rashidinejad H Sheikhvatan M. Diagnostic performance of electrocardiography in the assessment of significant coronary artery disease and its anatomical size in comparison with coronary angiography. J Res Med Sci. (2011) 16(6):750–5. Available online at: https://pmc.ncbi.nlm.nih.gov/articles/PMC3214392/pdf/JRMS-16-750.pdf 22091303 PMC3214392

[8] Global Burden of Cardiovascular Diseases and Risks 2023 Collaborators. Global, regional, and national burden of cardiovascular diseases and risk factors in 204 countries and territories, 1990–2023. J Am Coll Cardiol. (2025) 86(22):2167–243. 10.1016/j.jacc.2025.08.01 40990886

[9] Choi SH Lee H-G Park S-D Bae J-W Lee W Kim M-S. Electrocardiogram-based deep learning algorithm for the screening of obstructive coronary artery disease. BMC Cardiovasc Disord. (2023) 23(1). 10.1186/s12872-023-03326-4 PMC10246412 37286945

[10] Li R Zhao X Gong Y Zhang J Dong R Xia L. A new method for detecting myocardial ischemia based on ECG T-wave area curve (TWAC). Front Physiol. (2021) 12:660232. 10.3389/fphys.2021.660232 33868027 PMC8044312

[11] Braun T Spiliopoulos S Veltman C Hergesell V Passow A Tenderich G. Detection of myocardial ischemia due to clinically asymptomatic coronary artery stenosis at rest using supervised artificial intelligence-enabled vectorcardiography—a five-fold cross-validation of accuracy. J Electrocardiol. (2020) 59:100–5. 10.1016/j.jelectrocard.2019.12.018 32036110

[12] CONABIOS. Estudio piloto para evaluar la precisión diagnóstica de la Cardisiografía en el reconocimiento de la enfermedad cardiovascular CARDISIO-RD. Aprobación No. 03, mayo 17, 2023. (No. 034/2023). Available online at: https://conabios.gob.do/reglamento

[13] Lindner O Kammeier A Knobl H Tenderich K Burchert W. Vergleich der Cardisiographie (CSG) mit der Myokard-SPECT bei Verdacht auf KHK und bekannter KHK. Nuklearmedizin. (2022) 61:203. 10.1055/s-0042-1746136

[14] Aydar S Knobl H Burchert W Lindner O. Diagnostic accuracy of artificial intelligence-enabled vectorcardiography versus myocardial perfusion SPECT in patients with suspected or known coronary heart disease. Nuklearmedizin. (2024) 63(3):213–8. 10.1055/a-2263-2322 38378022 PMC11136534

[15] Rudland SV Shah NH Nevill A. Community-based cardiovascular risk assessment using the CARDISIOtm ai test: a prospective cohort study. BJGP Open. (2025) 9(3):BJGPO.2024.0183. 10.3399/bjgpo.2024.0183 40389278 PMC12728841

